# Understanding the dynamics of patient systems of implementation: a mixed methods study

**DOI:** 10.1186/1472-6963-14-S2-O11

**Published:** 2014-07-07

**Authors:** Anne Rogers, Ivo Vassilev, Anne Kennedy

**Affiliations:** 1 EU-WISE Team, University of Southampton, Southampton, UK

## Background

Compared to studies of professional implementation, patient systems of implementation remain under-investigated. Patient self-management for long term conditions has focused on the motivational and individual capacities in taking responsibility for managing well. Little attention has been focused on the collective resources work, and connections to others in patient health eco-systems as a potential means of effective self-management support and how these may vary in different cultural contexts.

The aim of this paper is to explore the work, meaning and function attributed to relationships and ties within personal communities of illness management. We will illuminate the properties of different members of networks in order to identify the place of these within a broader context of patient systems of support for long-term conditions.

## Materials and methods

A review of network properties and analysis relevant to 5 European countries (Bulgaria, Norway, Netherlands, Spain and Crete) and an indepth case study using mixed methods survey with nested qualitative study was performed. The latter is a UK based study of a total of 300 people from deprived areas in the North West of England with chronic illnesses conducted in 2010 to 2011. A research tool with which participants identified 2,544 network members who contributed to illness management was used to describe activities associated with chronic illness and to identify how ties are perceived to be involved through contributions of social network members.

## Results

The results provide an articulation of the types and properties of networks involved in chronic illness work. Weaker ties compared to stronger are fit for different purposes and may be more durable and less liable to loss over time. Navigation, negotiation and collective efficacy are core properties of self-managing networks. Work undertaken and accepted by those with a long term condition requires the acceptable moral positioning of the self-managing ‘self’ and a sense of reciprocity.

## Conclusions

A bridge between a sense of personal agency and control and the need for external support are inherent to patient systems of implementation. Access to weak tie resources need to be given more prominence in health services research and policy for long term conditions.

